# The impact of media reports on energy and environmental efficiency in China: evidence from modified dynamic DEA with undesirable outputs

**DOI:** 10.1186/s12962-021-00302-7

**Published:** 2021-08-16

**Authors:** Ying Li, Tai-Yu Lin, Yung-ho Chiu, Huaming Chen, Hongyi Cen

**Affiliations:** 1 Business School, Sichuan University, Wangjiang Road No. 29, Chengdu, 610064 People’s Republic of China; 2 Department of Business Administration, National Cheng Kung University, No. 1, University Road, Tainan City, 701 Taiwan, ROC; 3 Department of Economics, Soochow University, 56, Kueiyang St., Sec. 1, Taipei, 100 Taiwan, ROC; 4 Sichuan University, Wangjiang Road No. 29, Chengdu, 610064 People’s Republic of China

**Keywords:** Dynamic DEA, Energy, Efficiency, AQI, CO_2_, Media report

## Abstract

**Background:**

Rapid economic growth in China has resulted in a commensurate increase in energy consumption, which in turn has seen an increase in environmental pollution problems. Past research has tended to focus on energy and environmental efficiency analyses and has rarely examined the media influence on environmental protection efforts. Further, most studies have used static models and ignored the dynamic changes over time.

**Methods:**

To go some way to filling this research gap, this study developed a modified undesirable Dynamic DEA model that included air quality index (AQI) and CO_2_ indicators to explore the relationships between energy, the environment, and media report efficiencies in 31 Chinese cities from 2013 to 2016.

**Results:**

It was found that: (1) Chongqing, Guangzhou, Nanjing and Shanghai had efficiencies of 1, but Lanzhou, Shijiazhuang, Taiyuan, Xining and Yinchuan needed significant improvements; (2) Chongqing, Guangzhou, Kunming, Nanning and Shanghai had relatively high media efficiencies, but the other cities had low efficiencies and required improvements; (3) the CO_2_ emissions efficiencies in most cities were better than the air quality index efficiencies; and 4. the media reports in most cities were found to have a more positive impact on the CO_2_ emissions efficiency than on the AQI efficiency.

**Conclusions:**

Environmental awareness enhances civilian health and promotes economic growth. Therefore, as the news media should be responsible for promoting environmental protection, they need to increase their environmental pollution coverage. It was found that the environmental pollution media report quality and especially air pollution reports needed improvements, and greater media coverage on environmental pollution and awareness was needed.

## Background

The energy required for sustained economic development causes serious carbon dioxide, PM2.5, and sulfur dioxide emissions. The International Energy Agency (IEA) reported that in 2018, global carbon dioxide emissions reached 33,143 million tonnes, a 1.7% increase over the previous year and the highest growth rate since 2013. Because China’s carbon emissions accounted for 27% of the global total, it has now been the world’s largest greenhouse gas emitter for over a decade.

PM2.5 emissions have attracted significant attention. At the 2015 climate change conference, China established three goals in accordance with the Paris Agreement, one of which was a 60–65% reduction in carbon dioxide emissions by 2030. In response to these goals, the Chinese government issued commensurate policies, such as the air pollution prevention and control action plan and the Environmental Protection Law, and is implementing national emissions reduction targets.

As the media now has a wide range of access modes, it is followed by many people in China and could be used to encourage a national focus on reducing China’s environmental pollution problems.

The association between energy and environmental efficiency has been widely studied [1, 7–10, 13, 17, 26, 27, 29, 31, 34, 35, 36–40]. Some studies have examined the impact of energy efficiency on the overall efficiency of a country or region [2, 5, 16–18, 34–36], and others have explored the relationships between the mass media, air pollution, and environmental issues or awareness [3, 11, 14, 24, 25], with some particularly focusing on the effects of news media reports that improve the awareness of environmental protection issues [8, 21, 23, 25, 28, 37].

While past studies have examined the relationships between energy and environmental efficiency and between media reports and environmental awareness raising, there have been few discussions looking at the relationships between energy consumption, the environment, and the media. Further, as most studies have tended to employ static energy efficiency and environmental pollution analysis methods, there has been little consideration of dynamic sustainable energy consumption and environmental protection development over time. Therefore, to explore the relationships between energy consumption, media, and environmental efficiencies, this study developed a modified undesirable Dynamic data envelopment analysis (DEA) model that considered the dynamic effects of a carry-over and undesirable output. The air quality index (AQI) was included in the model analysis to more fully consider the relationships between energy consumption and environmental pollution. To avoid the shortcomings in static analysis and to assess the intertemporal influences, carry-over activities were also included in the inputs and outputs.

This paper makes two main contributions. First, in addition to exploring the energy and environmental efficiencies, media release factors were included in the model to comprehensively explore the relationships between energy consumption, the environment, and media communications. Second, to overcome the static analysis problems in previous studies, a dynamic carry-over effect was included in the modified undesirable Dynamic DEA model to examine the energy consumption, media, and environmental efficiencies in 31 Chinese cities from 2013 to 2016, with labor, energy consumption, and media reports as the inputs, GDP, CO_2,_ and AQI as the outputs, and fixed assets as the carry-over.

## Literature review

Past studies on the environment and mass media can be divided into three main categories: (1) energy and air pollution efficiency; (2) energy efficiency impacts on the overall efficiency of a country or region; and (3) the relationship between the mass media, air pollution, and environmental issues.

### Energy and air pollution efficiency

Air pollutant emissions mainly comes from the use of coal and oil-based energy sources. Therefore, environmental efficiency research needs to also focus on energy efficiency. The past studies reviewed in this section tended to focus on the relationships between energy use efficiency and air pollutant emissions in a country or region. For example, Hu and Wang [9] used a modified DEA model to analyze China’s energy efficiency, and found that China’s rapid economic development had improved overall energy efficiency, Yeh et al. [40] compared mainland Chinese and Taiwanese energy efficiencies using traditional DEA models, and Song et al. [31] used a Super-SBM model to measure and calculate the energy efficiencies in BRIC countries, finding that while all countries had lower energy efficiencies, they had faster growth trends. In other studies, Wang et al. [34, 35] used a multi-directional efficiency analysis (MEA) method to study regional energy and emissions efficiencies in China, finding that the eastern region was generally more efficient than the central and western regions, and Wang et al. [34, 35] studied CO_2_ emissions performances, the potential to reduce emissions in China’s provinces, and the impact of regulatory policies, finding that the CO_2_ emissions in the southeastern coastal area provinces were relatively high, and the CO_2_ emissions in the central and western inland areas were relatively low. Wu et al. [38] used a two-stage network DEA framework to assess Chinas energy conservation and emissions reduction efficiencies during China’s eleventh five-year plan (2006–2010), and found that: (1) China's eastern region had the best energy conservation and emissions reduction performances compared to the central and western regions, (2) the central region had better production efficiency than the western region, (3) the western region had more efficient processing than the central region. Li and Du [17] analyzed the impact of China's market-oriented reforms on China’s energy and carbon emissions efficiency, and found that while the energy use and carbon dioxide emissions performances were poor in most parts of China, the eastern provinces generally performed better than the central and western regions. Meng et al. [26] conducted comprehensive survey studies from 2006 to 2015 that had used DEA models to assess China’s EE&CE (energy and carbon discharge efficiency) in 30 provinces or regions from 1995 and 2012, finding that eastern China had the highest EE&CE, and central China had the lowest. Yao et al. [39] used panel data and a meta-frontier non-radial Malmquist CO_2_ emissions performance index (MNMCPI) to assess China’s provincial industrial sector from 1998 to 2011 and estimate China’s CO_2_ emissions efficiency, finding that the average annual CO_2_ emissions growth rate in China’s provincial industrial sector was 5.53% from 1998 to 2011. Jebali et al. [13] studied the energy efficiencies in Mediterranean countries from 2009–2012, and found that the they were very high. Abbas et al. [1] reviewed the use of DEA models for energy efficiency development, and Qin et al. [29] used DEA to assess the energy efficiency of China’s coastal areas from 2000 to 2012, and found that economic development levels were positively correlated with energy efficiencies, and the Bohai Economic Zone had improved energy efficiency and greater air emissions. In other energy and environmental efficiency studies, Feng et al. (2017) found that China’s CO_2_emissions were inefficient, Hu et al. [10] used a congestion total-factor energy efficiency model to analyze the energy efficiencies in 20 Taiwanese administrative regions from 2004 to 2013, and Mehmeti et al. [27] analyzed high temperature fuel cells (HTFC) for power-gas relationships.

### Impact of energy efficiency on the overall efficiency of a country or region

Energy use can promote the development of a country or region. The studies reviewed in this section focus on the impact of energy efficiency on overall and technical efficiencies. Bampatsou et al. [2] used DEA to analyze the technical efficiencies in 15 EU countries from 1980 to 2008, and found that the integration of nuclear energy in the energy mix had had a negative impact, Wang et al. [34, 35] extended a directional distance function (DDF) using stochastic front analysis techniques to evaluate the CO_2_ emissions efficiencies in various Chinese provinces, and found that the southeastern coastal areas had higher CO_2_ emissions, and Li et al. [16] used a DEA-Malmquist method to analyze the energy intensity in China from 2000 to 2009, finding that China’s energy intensity could be reduced based on three internal influencing factors,economic structure, energy consumption structure, and technological progress. In other studies, Lin and Du [17] used a non-radial DDF to explore regional energy and CO_2_ emissions efficiency in China from 1997 to 2009, and found that China’s energy use and CO_2_ emissions performances were poor, with the eastern provinces generally performing better than the central and western regions, Li and Lin [18] proposed a total-factor energy consumption performance index (TEPI) and used a two-stage double bootstrap approach to measure China’s energy efficiency from 1997 to 2012, and found that China’s energy technology innovations had a negative impact on TEPI, and Du et al. [5] used DEA to analyze China’s CO_2_ emissions from 2006 to 2012 and conduct a cross-provincial comparison. In more recent studies, Wang et al. [36] using stochastic frontier analysis to analyze China’s energy productivity from 1995 to 2012, and concluded that the overall change in energy productivity had been mainly affected by the steady positive and negative technological progress change rates.

### Relationship between the mass media, air pollution, and the environment

The media plays an important role in disseminating information to the public about the health problems associated with air pollution and the severity of environmental pollution to enhance general environmental awareness. This section reviews studies on the relationship between the mass media, air pollution and environmental issues. In an early study, Lowe and Morrison [21] studied the role of the media in popularizing environmental issues, and later McCreery [25] believed that media attention was a predictor of air pollution and was more important than environmental policies, Niklas et al. [28] found that public environmental issues were affected by the number of media reports, and Mayer [24] found that the lack of coherence and specificity of the media possibly undermined the ability of regulators to reform air pollution policy. Mason [23] specifically examined the effectiveness of corporate environmental reporting by 100 companies in promoting social responsibility in groups such as employees and shareholders and outside members such as consumers, Chen [3] analyzed reports from China’s official English-language newspaper, the China Daily, on air pollution in China in 2013, and Wang et al. [37] studied Chinese social media to monitor air quality trends and related public perceptions and responses, finding that the information on Weibo reflected the true level of particle pollution. Kay et al. [14] took Sina Weibo as a research object and concluded that it effectively promoted public environmental awareness, Foxwell-Norton and Konkes [8] found that the Australian news media played an important role in protecting coral reefs, and Hswen et al. [11] found that social media could aid in environmental monitoring.

However, in most of these studies, there was little focus on the relationships between the media and AQI and most employed static analyses that did not consider any carry-over effects. Therefore, to address these research gaps, this paper developed a modified Undesirable Dynamic DEA model to explore the energy, media, and environmental efficiencies in 31 Chinese cities.

## Research method

The dynamic DEA concept was first proposed by Klopp [15] based on Malmquist [22] and then extended by Fare et al. [6]. However, while these models measured changes in intertemporal efficiency, they did not consider the effects of the intertemporal continuation activities and therefore, were less suitable for measuring long-term efficiencies. Tone and Tsutsui [32] then included a carry-over and extended the slacks-based measure model (SBM) to a dynamic analysis. The model description is as follows (without undesirable variable),

Suppose the observation is a $$J$$(J = 1…n) dimension decision making unit (DMU) set in which the DMU under evaluation is represented by $$DMU_{O}$$ and subject to $$DMU_{O}$$$$\in J$$, with inputs and outputs used to compute the efficiency labeled m inputs $$x_{ijt}$$ (i = 1…m) and s outputs $${\text{Y}}_{ljt}$$ with $${Z}^{good}$$ carried over from period t to period t + 1. The following is the non-oriented model:

1 $$\theta_{0}^{*} = \min \frac{{\frac{1}{T}\sum\nolimits_{t = 1}^{T} {W^{t} \left[ {1 - \frac{1}{m + ninput}\left[ {\sum\limits_{i = 1}^{m} {\frac{{s_{it}^{ - } }}{{x_{iot} }} + \sum\limits_{r = 1}^{nbad} {\frac{{s_{rt}^{input} }}{{z_{rot}^{input} }}} } } \right]} \right]} }}{{\frac{1}{T}\sum\nolimits_{t = 1}^{T} {W^{t} } \left[ {1 + \frac{1}{{s_{1} }}\left[ {\sum\limits_{l = 1}^{{s_{1} }} {\frac{{s_{jt}^{ + g} }}{{y_{lot}^{g} }}} } \right]} \right]}}$$

Equation (1) is the connection equation between t and t + 1.

$$\sum_{\mathrm{j}=1}^{\mathrm{n}}{\mathrm{z}}_{\mathrm{ijt}}^{\mathrm{\alpha }}{\uplambda }_{\mathrm{j}}^{\mathrm{t}}=\sum_{\mathrm{j}=1}^{\mathrm{n}}{\mathrm{z}}_{\mathrm{ijt}}^{\mathrm{\alpha }}{\uplambda }_{\mathrm{j}}^{\mathrm{t}+1} \left(\forall \mathrm{i};\mathrm{t}=1,\dots ,\mathrm{T}-1\right),$$

$${\mathrm{x}}_{\mathrm{iot}}=\sum_{j=1}^{n}{\uplambda }_{i}^{t}+{s}_{it}^{-} \left(\mathrm{i}=1,\dots ,\mathrm{m};\mathrm{t}=1,\dots ,\mathrm{T}\right),$$

$${\mathrm{y}}_{\mathrm{lot}}=\sum_{\mathrm{j}=1}^{\mathrm{n}}{y}_{ljt}^{+g}{\uplambda }_{\mathrm{j}}^{\mathrm{t}}-{\mathrm{s}}_{\mathrm{lt}}^{+} \left(\mathrm{l}=1,\dots ,\mathrm{s}1;\mathrm{t}=1,\dots ,\mathrm{T}\right),$$

$${\mathrm{Z}}_{\mathrm{rot}}^{\mathrm{input}}=\sum_{\mathrm{j}=1}^{\mathrm{n}}{\mathrm{z}}_{\mathrm{rjt}}^{\mathrm{input}}{\uplambda }_{\mathrm{j}}^{\mathrm{t}}+{\mathrm{s}}_{\mathrm{rt}}^{\mathrm{input}} \left(\mathrm{r}=1,\dots ,\mathrm{ninput};\mathrm{t}=1,\dots ,\mathrm{T}\right),$$

$$\sum_{\mathrm{j}=1}^{\mathrm{n}}{\uplambda }_{\mathrm{j}}^{\mathrm{t}} =1 \left(\mathrm{t}=1,\dots ,\mathrm{T}\right),$$

2 $${\uplambda }_{\mathrm{j}}^{\mathrm{t}}\ge 0, {\mathrm{s}}_{\mathrm{it}}^{-}\ge 0, {\mathrm{s}}_{\mathrm{lt}}^{+}\ge 0.$$

The most efficient solution is:

3 $$\theta_{0}^{*} = \min \frac{{\left[ {1 - \frac{1}{m + ninput}\left[ {\sum\limits_{i = 1}^{m} {\frac{{s_{it}^{ - } }}{{x_{iot} }} + \sum\limits_{r = 1}^{nbad} {\frac{{s_{rt}^{input} }}{{z_{rot}^{input} }}} } } \right]} \right]}}{{1 + \frac{1}{{s_{1} }}\left[ {\sum\limits_{l = 1}^{{s_{1} }} {\frac{{s_{jt}^{ + g} }}{{y_{lot}^{g} }}} } \right]}}$$

### Modified undesirable dynamic SBM model

However, the above model does not consider undesirable outputs. As this study used panel data from 31 of the most developed cities across China, it was assumed that there was a consistent and homogenous balance. Six variables were employed: three inputs; employees, energy consumption and media reports; three outputs; GDP (desirable) CO_2_ (undesirable) and AQI (undesirable); and one carry-over; fixed assets.

The reduction of CO_2_ emissions is one of the most important tasks in the global climate change. At the climate conference, all countries pledged to control carbon dioxide emissions within a certain range. Therefore, the CO_2_ emissions in this study are undesirable output. AQI is the Air Quality Index. AQI describes the degree of air cleanliness or pollution, and is a new air quality evaluation standard issued by China in March 2012. There are six pollutant monitoring items: sulfur dioxide, nitrogen dioxide, PM10, PM2.5, carbon monoxide and ozone. The data is updated every hour. AQI presents the pollution degree of these 6 pollutants by using a unified evaluation standard. Therefore, this study regards AQI as an undesirable output. CO_2_ and AQI have a bad effect on production and both CO_2_ and AQI are Non Separable bad output.

Because the undesirable outputs (CO_2_ and AQI) are considered in the dynamic SBM model, Tone and Tsutsui’s [32] dynamic SBM model was modified to include undesirable output.

The observation was a *J*(J = 1….n) dimension decision making unit (DMU) set in which the DMU under evaluation was represented by *DMU*_*o*_ and subject to *DMU*_*o*_ ϵ J. The inputs and outputs used to compute the efficiency were labeled as m inputs *x*_*ijt*_ (i = 1…m) and s outputs *Y*_*ljt*_. Output Y was divided into (Y^g^, Y^b^), where Y^g^ was the desirable output, Y^b^ was the undesirable output, and *z*^*input*^ was the carryover from period t to period t + 1. The following is the developed non-oriented model:

4 $$\theta^{t}_{\mathrm{o}}={\rm min}\frac{\frac{1}{T}\sum_{t=1}^{T}{W^{t}} {\left[ {1 - \frac{1}{m + ninput}\left[ {\sum\limits_{i = 1}^{m} {\frac{{s_{it}^{ - } }}{{x_{iot} }} + \sum\limits_{r = 1}^{nbad} {\frac{{s_{rt}^{input} }}{{z_{rot}^{input} }}} } } \right]} \right]}}{{\frac{1}{T}\sum_{t=1}^{T}{W^{t}}\left[{1 + \frac{1}{{s_{1} }}\left[ {\sum\limits_{l = 1}^{{s_{1} }} {\frac{{s_{jt}^{ + g} }}{{y_{lot}^{g} }}}+\sum\limits_{l = 1}^{{s_{1} }} {\frac{{s_{jt}^{ - b} }}{{y_{lot}^{b} }}} } \right]}\right]}}$$

Equation (4) was the connection equation between t and t + 1.

$$\sum_{\mathrm{j}=1}^{\mathrm{n}}{\mathrm{z}}_{\mathrm{ijt}}^{\mathrm{\alpha }}{\uplambda }_{\mathrm{j}}^{\mathrm{t}}=\sum_{\mathrm{j}=1}^{\mathrm{n}}{\mathrm{z}}_{\mathrm{ijt}}^{\mathrm{\alpha }}{\uplambda }_{\mathrm{j}}^{\mathrm{t}+1} \left(\forall \mathrm{i};\mathrm{t}=1,\dots ,\mathrm{T}-1\right),$$

$${\mathrm{x}}_{\mathrm{iot}}=\sum_{i=1}^{m}{\uplambda }_{i}^{t}+{s}_{it}^{-} \left(\mathrm{i}=1,\dots ,\mathrm{m};\mathrm{t}=1,\dots ,\mathrm{T}\right),$$

5 $${\mathrm{y}}_{\mathrm{lot}}=\sum_{\mathrm{l}=1}^{\mathrm{s}1}{y}_{lot}^{+g}{\uplambda }_{\mathrm{j}}^{\mathrm{t}}-{\mathrm{s}}_{\mathrm{lt}}^{+\mathrm{g}} \left(\mathrm{l}=1,\dots ,\mathrm{s}1;\mathrm{t}=1,\dots ,\mathrm{T}\right),$$

$${\mathrm{y}}_{\mathrm{lot}}=\sum_{\mathrm{l}=1}^{\mathrm{s}2}{y}_{lot}^{-b}{\uplambda }_{\mathrm{j}}^{\mathrm{t}}+{\mathrm{s}}_{\mathrm{lt}}^{-b} \left(\mathrm{l}=1,\dots ,\mathrm{s}2;\mathrm{t}=1,\dots ,\mathrm{T}\right),$$

$${\mathrm{z}}_{\mathrm{rot}}^{\mathrm{input}}=\sum_{\mathrm{r}=1}^{\mathrm{n}}{\mathrm{z}}_{\mathrm{rjt}}^{\mathrm{input}}{\uplambda }_{\mathrm{j}}^{\mathrm{t}}+{\mathrm{s}}_{\mathrm{rt}}^{\mathrm{input}} \left(\mathrm{r}=1,\dots ,\mathrm{ninput};\mathrm{t}=1,\dots ,\mathrm{T}\right),$$

$$\sum_{\mathrm{j}=1}^{\mathrm{n}}{\uplambda }_{\mathrm{j}}^{\mathrm{t}} =1 \left(\mathrm{t}=1,\dots ,\mathrm{T}\right),$$

6 $${\uplambda }_{\mathrm{j}}^{\mathrm{t}}\ge 0,{\mathrm{s}}_{\mathrm{it}}^{-}\ge 0, {\mathrm{s}}_{\mathrm{lt}}^{+\mathrm{g}}\ge 0,{, {\mathrm{s}}_{\mathrm{lt}}^{-b}\ge 0,\mathrm{ s}}_{\mathrm{rt}}^{\mathrm{good}}\ge 0.$$

The most efficient solution, therefore, is:

7 $${\uprho }_{0\mathrm{t}}=\frac{{1 - \frac{1}{m + ninput}\left[ {\sum\limits_{i = 1}^{m} {\frac{{s_{it}^{ - } }}{{x_{iot} }} + \sum\limits_{r = 1}^{nbad} {\frac{{s_{rt}^{input} }}{{z_{rot}^{input} }}} } } \right]} }{{1 + \frac{1}{{s_{1} }}\left[ {\sum\limits_{l = 1}^{{s_{1} }} {\frac{{s_{jt}^{ + g} }}{{y_{lot}^{g} }}}+\sum\limits_{l = 1}^{{s_{1} }} {\frac{{s_{jt}^{ - b} }}{{y_{lot}^{b} }}} } \right]}} \left(\mathrm{i}=1,\dots ,\mathrm{T}\right).$$

### Labor, energy consumption, media report, GDP, CO_2_ and AQI efficiencies

Hu and Wang’s (2006) total-factor energy efficiency index was employed to overcome any possible bias in the traditional energy efficiency indicators. There were six key variables in this study: labor, energy consumption, media reports, GDP, CO_2_, and AQI, with “I” representing area and “t” representing time.

The six efficiency models were therefore defined as follows:

8 $${\text{Labor efficiency}}= {\frac{{\text{Target Labor input (i, t)}}}{{\text{Actual Labor input (i, t)}}}}$$

9 $${\text{Energy consumption efficiency}}= \frac{{\text{Target Energy input (i, t)}}}{{\text{Actual energy input(i, t)}}}$$

10 $${\text{Media efficiency}}= \frac{{\text{Target media input (i, t)}}}{{\text{Actual media input (i, t)}}}$$

11 $${\text{GDP}}\;{\text{efficiency}} = \frac{{{\text{Actual}}\;{\text{GDP}}\;{\text{desirable}}\;{\text{output}}\;{\text{(i,t)}}}}{{{\text{Target}}\;{\text{GDP}}\;{\text{desirable}}\;{\text{output}}\;{\text{(i,t)}}}}$$

12 $${\text{CO}}_{2} \;{\text{efficiency}} = \frac{{{\text{Target}}\;{\mathbf{CO2}}\;{\text{undesirable}}\;{\text{output}}\;{\text{ }}({\text{i, t}})}}{{{\text{Actual}}\;{\mathbf{CO2}}\;{\text{undesirable}}\;{\text{output}}\;{\text{ }}({\text{i, t}})}}$$

13 $${\text{AQI efficiency}} =\frac{{\text{Target AQI undesirable output (i, t)}}}{{\text{Actual AQI undesirable output (i, t)}}}$$

If the target labor, energy consumption, and media input equaled the actual input, then the labor, energy consumption and media efficiencies were 1, indicating overall efficiency. If the target labor, energy consumption, and media input were less than the actual input, then the labor, energy consumption, and media efficiencies were less than 1, indicating overall inefficiency.

If the target GDP desirable output was equal to the actual GDP desirable output, then the GDP efficiency was 1, indicating overall efficiency. If the actual GDP desirable output was less than the target GDP desirable output, then the GDP efficiency was less than 1, indicating overall inefficiency.

If the target undesirable CO_2_ and AQI output efficiencies were equal to the actual undesirable CO_2_ and AQI output efficiencies, then the CO_2_ and AQI efficiencies equaled 1, and were efficient. If the target CO_2_ and AQI undesirable output efficiencies were less than the actual CO_2_ and AQI undesirable outputs, then the CO_2_ and AQI efficiencies were less than 1, indicating inefficiency.

## Empirical study

### Data sources

2013 to 2016 panel data from 31 of the most developed cities from eastern to western China were extracted from the Statistical Yearbooks of China, the Demographics and Employment Statistical Yearbooks of China, and each city’s Statistical Yearbooks. The air pollutant data were collected from the China Environmental and Protection Bureau Annual Reports and the China Environmental Statistical Yearbook.

**Fig. 1:**
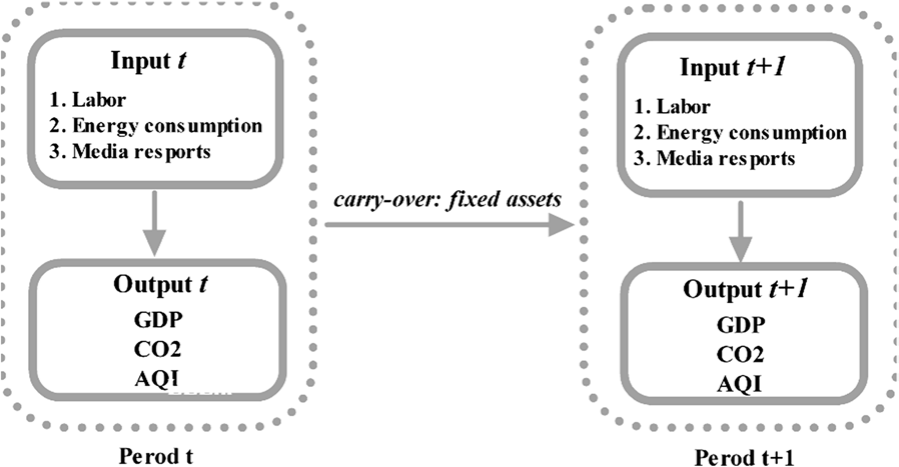
Efficiency analysis for economic growth, carbon emissions and air pollutant emissions under the influence of news media

As the 31 cities vary in population, industrial aggregation, natural resources, meteorological conditions, and geography, as a whole they represented the general air pollution emissions and treatment situation in China. As in past research on energy and the environment, the inputs were labor, energy consumption, fixed assets, and media reports [5, 16, 35], and the outputs were GDP as the desirable output, and CO_2_ and AQI as the undesirable outputs. Past studies have included CO_2_ emissions and ignored AQI indicators [12, 30], however, as China regarded the Air Pollution Control Action Plan as one of its economic development considerations in 2013, only including CO_2_ as the undesirable output and ignoring other undesirable air pollutants such as PM_2.5_, SO_2_, and NO_2_ could lead to biased reports and poor policy recommendations. Therefore, as in several previous studies, both CO_2_ and the AQI air pollution indicators are included in the model analysis [4, 19, 20, 33–35, 40]. This study deferred the production process through fixed assets to the second phase, and analyzed the efficiency of economic growth (GDP), carbon emissions (CO_2_) and air pollutant emissions (AQI) as well as accounting for the news media input, as shown in Fig. 1.

The variables using in this study are explained in Table 1 and defined as follows.

**Table 1:** Input and output variables

Input variables	Output variables	Carry over
Labor	GDP	Fixed assets
Energy consumption	CO_2_	
Media reports	AQI	

#### Input variables

Labor input: unit = people; the number of employees in each city at the end of each year.

Energy consumption: unit = 100 million tonnes; the total energy consumption in each city each year.

Media reports: news or press releases related to “province + air pollution”, which were extracted from The People’s Daily and Xinhuanet Media’s official website.

#### Output variables

##### Desirable output index

GDP in each city; unit = 100 million CNY.

##### Undesirable output index

CO_2_ and Air quality Index (AQI), which was the measured concentration of air pollutants ad incorporated particulate matter (PM_2.5_, PM_10_), sulfur dioxide (SO_2_) and Nitrogen.

##### Carry over index

Fixed assets: unit: 100 million CNY, which was measured as the capital stock in each city at the end of each year.

#### Statistical analysis

**Fig. 2:**
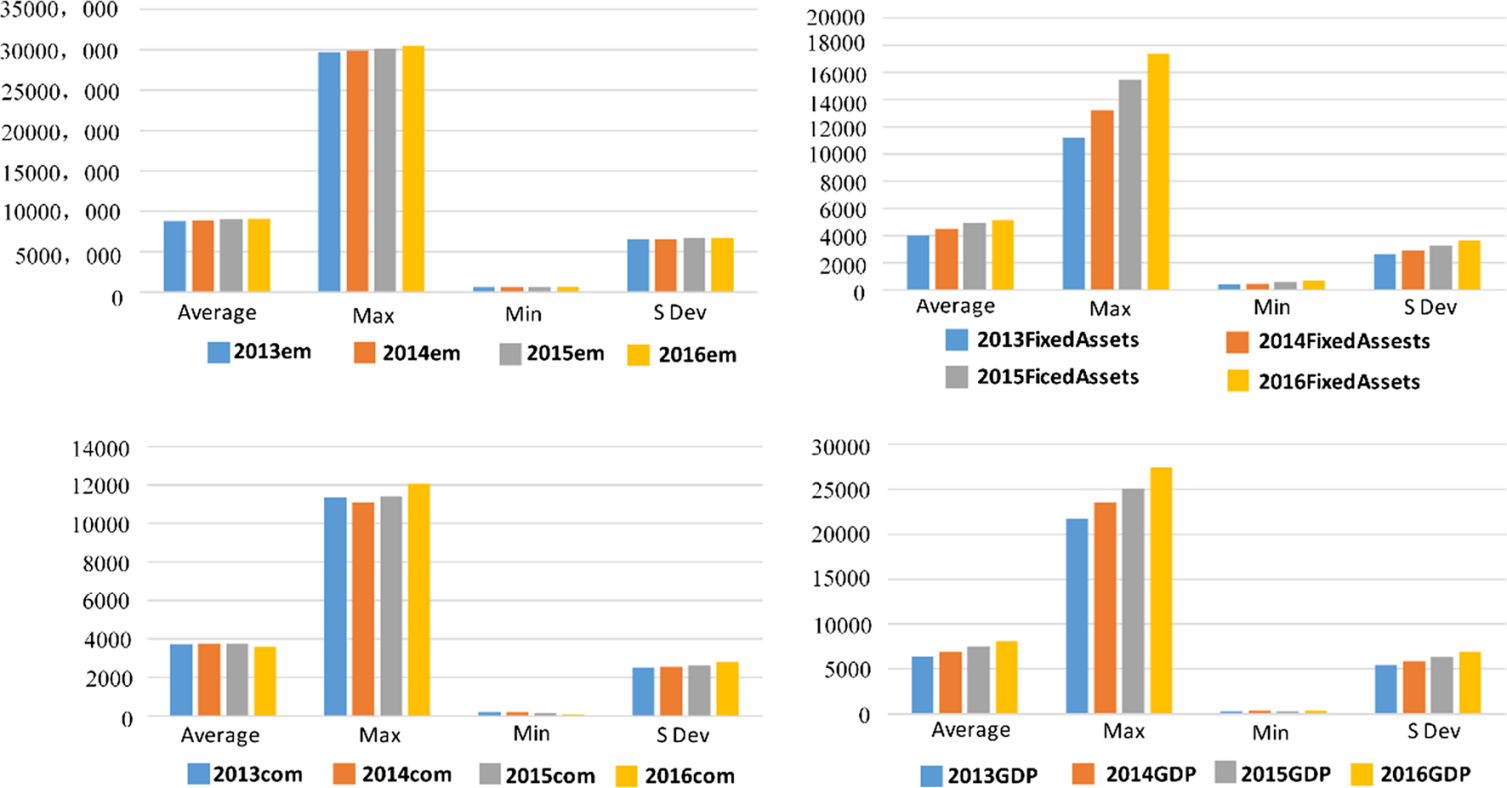
Labor, fixed assets, energy consumption and GDP

Figure 2 shows the overall labor, fixed assets investment, energy consumption input and GDP output indicators from the 31 cities in China from 2013 to 2016. It can be seen that there was substantial growth in both the fixed assets and GDP inputs, with rises also being seen also in the maximum, average, and minimum values. The labor minimum, maximum, and average values rose slightly, which may have been related to the decline in population growth in China during this period. The maximum energy consumption fell to its lowest level in 2014 and then rebounded to its highest level in 2016; however, the average and minimum values continued to decline.

**Fig. 3:**
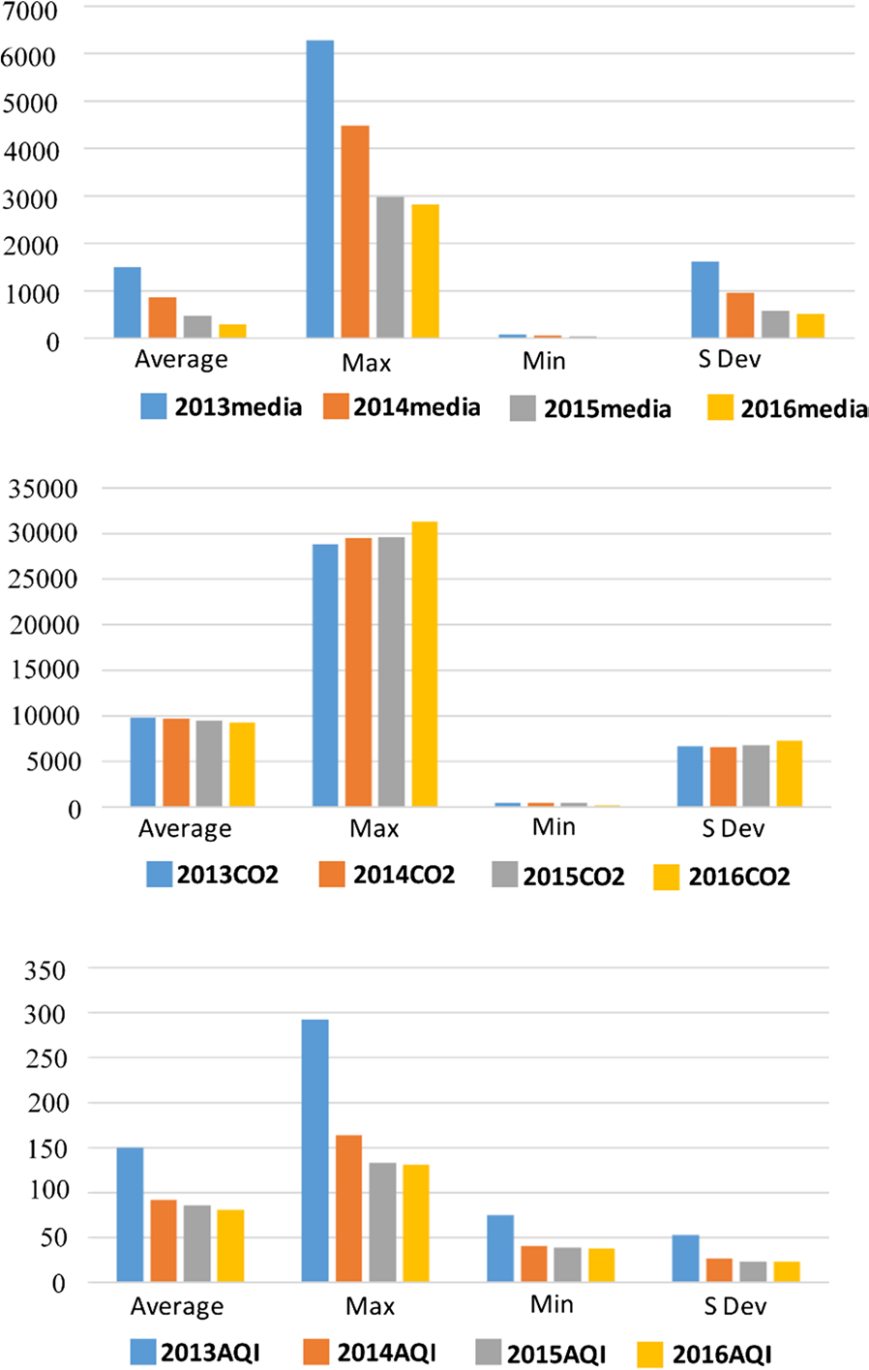
News media, CO_2_ and AQI statistics

Figure 3 shows the changes in the number of associated news media reports, CO_2_ emissions and the AQI from 2013 to 2016, from which it can be seen that the news media reports’ maximum, minimum and average dropped significantly, with the maximum reaching its highest level in 2013 and its lowest level in 2016, and with the average and minimum values showing the same trends. Reports on air pollution decreased, which may also reflect the public’s lower concerns about air pollution. The maximum CO_2_ emissions rose slightly, the minimum and average values decreased slightly, and the gap between the maximum and minimum widened.

### Results

#### Overall efficiency

As can be seen in Table 2, from 2013 to 2016, the average efficiencies in the 31 cities varied, with most cities needing improvements. The overall efficiency was 1 in Chongqing, Guangzhou, Nanjing and Shanghai, and Beijing’s efficiency was high in the first three years, but by 2016 had fallen to 0.6572. The total efficiencies in Changsha, Guiyang, Haikou, Hangzhou, Hefei, Huhehot, Lhasa, Nanchang, Shenyang, Tianjin, Wuhan, Urumqi and Zhengzhou ranged from 0.4 to 0.7, and in Lanzhou, Shijiazhuang, Taiyuan, Xining and Yinchuan were the worst, with the highest efficiency not exceeding 0.4.

**Table 2:** Annual overall efficiencies in 31 Chinese cities from 2013 to 2016

No.	City	2013	2014	2015	2016
1	Beijing	1.0000	1.0000	1.0000	0.6572
2	Changchun	0.6605	0.7283	0.5380	0.5798
3	Changsha	0.5716	0.4837	0.4817	0.5052
4	Chengdu	1.0000	0.9353	0.7527	0.7289
5	Chongqing	1.0000	1.0000	1.0000	1.0000
6	Fuzhou	0.7017	0.5871	0.5372	0.5310
7	Guangzhou	1.0000	1.0000	1.0000	1.0000
8	Guiyang	0.5288	0.5546	0.5564	0.5386
9	Harbin	0.7303	0.8016	0.5205	0.5229
10	Haikou	0.6274	0.5166	0.4795	0.6234
11	Hangzhou	0.5889	0.5369	0.5210	0.5609
12	Hefei	0.4867	0.4744	0.4586	0.4657
13	Huhehot	0.5686	0.5324	0.6147	0.5608
14	Jinan	0.3959	0.3946	0.4252	1.0000
15	Kunming	0.6139	0.6060	0.6949	0.7064
16	Lanzhou	0.3362	0.3344	0.3000	0.3001
17	Lhasa	0.6308	0.5204	0.4840	0.6040
18	Nanchang	0.5019	0.4876	0.4456	0.4458
19	Nanjing	0.3920	0.4478	0.4988	0.5019
20	Nanning	1.0000	1.0000	1.0000	1.0000
21	Shanghai	1.0000	1.0000	1.0000	1.0000
22	Shenyang	0.6182	0.6326	0.5003	0.6422
23	Shijiazhuang	0.3665	0.3588	0.3651	0.3482
24	Taiyuan	0.3471	0.3561	0.3413	0.3092
25	Tianjin	0.6600	0.5658	0.5154	0.5486
26	Wuhan	0.6552	0.5799	0.5606	0.5324
27	Urumqi	0.4294	0.4298	0.4406	0.4371
28	Xian	1.0000	0.8713	0.8277	0.7607
29	Xining	0.2768	0.2900	0.2703	0.3988
30	Yinchuan	0.3511	0.3311	0.2934	0.2764
31	Zhengzhou	0.6317	0.5207	0.5340	0.5959

The overall efficiencies in Jinan, Kunming, Nanjing, and Shenyang increased, with the largest increase being in Jinan from around 0.4 in 2013 and 2014 to 1 in 2016, and the largest fall in efficiency being in Beijing. The efficiencies in Changchun and Harbin increased from 2013 to 2014, but decreased in the last two years. The efficiencies in Chengdu and Xi’an both fell from 1 in 2013 to less than 0.8 in 2016.

#### Input and output efficiency indicators

Table 3 shows the media report efficiencies in the 31 cities from 2013 to 2016, from which it can be seen that a downward efficiency trend was more obvious than an upward trend. Chongqing, Guangzhou, Kunming, Nanning, and Shanghai had media efficiencies of 1, Xi’an had a media efficiency of 0.9544 in 2015, and 1 in all other years, Beijing’s media efficiency was 1 from 2013 to 2015, but only 0.0888 in 2016, Hefei, Lanzhou, Nanchang, Nanjing, Shijiazhuang, Urumqi, Xining, and Yinchuan had lower media efficiencies, Lanzhou, Shijiazhuang, Xining, Yinchuan and Urumqi’s media report efficiencies were below 0.4, and the lowest efficiency was in Nanning at below 0.3.

**Table 3:** Media report efficiencies

No.	DUM	2013 Media	2014 Media	2015 Media	2016 Media
1	Beijing	1.0000	1.0000	1.0000	0.0888
2	Changchun	1.0000	1.0000	0.4530	0.5095
3	Changsha	0.7947	0.2940	0.2613	0.3511
4	Chengdu	1.0000	1.0000	0.6895	0.7018
5	Chongqing	1.0000	1.0000	1.0000	1.0000
6	Fuzhou	1.0000	0.5194	0.2958	0.2476
7	Guangzhou	1.0000	1.0000	1.0000	1.0000
8	Guiyang	1.0000	1.0000	0.7661	0.6939
9	Harbin	1.0000	1.0000	0.3266	0.3145
10	Haikou	1.0000	0.5106	0.3486	1.0000
11	Hangzhou	0.7641	0.2972	0.1757	0.2636
12	Hefei	0.5486	0.2558	0.1693	0.1610
13	Huhehaote	1.0000	0.6771	0.8171	0.6576
14	Jinan	0.1230	0.2002	0.2545	1.0000
15	Kunming	1.0000	1.0000	1.0000	1.0000
16	Lanzhou	0.3103	0.3244	0.2357	0.1733
17	Lhasa	1.0000	0.4733	0.3449	1.0000
18	Nanchang	0.4642	0.2935	0.1600	0.3062
19	Nanjing	0.1958	0.2582	0.1836	0.1670
20	Nanning	1.0000	1.0000	1.0000	1.0000
21	Shanghai	1.0000	1.0000	1.0000	1.0000
22	Shenyang	1.0000	1.0000	0.4420	0.9326
23	Shijiazhuang	0.3354	0.2719	0.2119	0.2302
24	Taiyuan	0.6766	0.7599	0.6630	0.4026
25	Tianjin	1.0000	0.7134	0.5127	0.5948
26	Wuhan	1.0000	0.5060	0.3925	0.2920
27	Wulumuqi	0.1846	0.2157	0.1367	0.3238
28	Xian	1.0000	1.0000	0.9544	1.0000
29	Xining	0.2904	0.3670	0.2216	0.2259
30	Yinchuan	0.3333	0.2819	0.2000	0.1148
31	Zhengzhou	0.4463	0.1225	0.0942	0.8728

The media report efficiency was only rising in Jinan from 0.1 in 2013 to 1 in 2016, and the efficiency in Lhasa was 1 in 2013 to 2016 but in the middle years was less than 0.5. All other 23 cities had falling media report efficiencies, with the largest decline being in Wuhan from 1 in 2013 to 0.3 in 2016. Therefore, the media report efficiencies in most cities had little impact on environmental governance.

Table 4 shows the energy consumption efficiencies in the 31 Chinese cities from 2013 to 2016. Beijing, Chongqing, Guangzhou, Nanning, Shanghai had energy consumption efficiencies of 1, Chengdu, Lhasa, Haikou, Hefei and Xi’an all attained 1 in three years, Changsha, Lanzhou, Shijiazhuang, Tianjin and Yinchuan had lower efficiencies, and the lowest energy consumption efficiency was in Taiyuan with all years below 0.3.

**Table 4:** Energy consumption efficiencies

No.	DUM	2013 com	2014 com	2015 com	2016 com
1	Beijing	1.0000	1.0000	1.0000	1.0000
2	Changchun	0.9000	1.0000	0.9629	0.9772
3	Changsha	0.5937	0.7051	0.7119	0.6986
4	Chengdu	1.0000	0.8444	1.0000	1.0000
5	Chongqing	1.0000	1.0000	1.0000	1.0000
6	Fuzhou	0.9534	0.9486	0.9783	0.9748
7	Guangzhou	1.0000	1.0000	1.0000	1.0000
8	Guiyang	0.5263	0.6662	0.8048	0.9157
9	Harbin	0.9684	0.9994	0.8697	0.8388
10	Haikou	1.0000	0.9326	1.0000	1.0000
11	Hangzhou	0.6541	0.8137	0.8612	0.8664
12	Hefei	0.7951	0.9984	1.0000	1.0000
13	Huhehaote	0.4190	0.8416	0.8415	0.8026
14	Jinan	0.6914	0.4810	0.5829	1.0000
15	Kunming	0.5398	0.6160	0.8412	0.8496
16	Lanzhou	0.5757	0.5676	0.4725	0.5080
17	Lhasa	1.0000	0.9100	1.0000	1.0000
18	Nanchang	0.9134	0.9957	0.9731	0.8402
19	Nanjing	0.5853	0.5729	0.8323	0.8224
20	Nanning	1.0000	1.0000	1.0000	1.0000
21	Shanghai	1.0000	1.0000	1.0000	1.0000
22	Shenyang	0.6613	0.6134	0.7766	0.9030
23	Shijiazhuang	0.6944	0.7436	0.6312	0.6940
24	Taiyuan	0.2522	0.2668	0.2607	0.3127
25	Tianjin	0.6323	0.6352	0.6483	0.6683
26	Wuhan	0.7425	0.8115	0.8543	0.8427
27	Wulumuqi	0.8149	0.8573	0.9303	0.7825
28	Xian	1.0000	0.8932	0.9999	1.0000
29	Xining	0.4186	0.4207	0.4428	0.9703
30	Yinchuan	0.5295	0.4735	0.3949	0.3796
31	Zhengzhou	0.9966	0.9138	1.0000	0.5152

Changchun, Changsha, Fuzhou, Guiyang, Hangzhou, Hefei, Hohhot, Kunming, Nanjing, Shenyang, Taiyuan, Tianjin, Wuhan, and Xining had rising energy consumption efficiencies, and Harbin, Lanzhou, Nanchang, Urumqi, Yinchuan, and Zhengzhou had falling energy consumption efficiencies.

Table 5 indicates that the GDP efficiencies were high in most cities, with 17 of the 31 cities having efficiencies greater than 0.8. Beijing, Changchun, Guangzhou, Chongqing, Nanning, and Shanghai all had GDP efficiencies of 1, Harbin and Zhengzhou had efficiencies of 1 in three years, and Nanjing, Tianjin, and Nanchang had efficiencies of 1 for two years. Fuzhou, Haikou, Hohhot, Lanzhou, Lhasa, Shijiazhuang, Taiyuan, Urumqi, Xining and Yinchuan, Lanzhou, Shijiazhuang and Xining had the lowest GDP efficiencies, with Fuzhou, Shijiazhuang, Taiyuan, Urumqi, Xining, and Yinchuan all having fluctuating efficiencies.

**Table 5:** GDP efficiencies

No.	DMU	2013 GDP	2014 GDP	2015 GDP	2016 GDP
1	Beijing	1.0000	1.0000	1.0000	1.0000
2	Changchun	1.0000	1.0000	1.0000	1.0000
3	Changsha	1.0000	0.8790	0.8816	0.8892
4	Chengdu	1.0000	1.0000	0.9082	0.8524
5	Chongqing	1.0000	1.0000	1.0000	1.0000
6	Fuzhou	0.7228	0.7159	0.7169	0.7226
7	Guangzhou	1.0000	1.0000	1.0000	1.0000
8	Guiyang	0.8144	0.7488	0.6772	0.6585
9	Harbin	0.9223	1.0000	1.0000	1.0000
10	Haikou	0.6923	0.6939	0.6755	0.6694
11	Hangzhou	1.0000	0.8637	0.8686	0.8782
12	Hefei	1.0000	0.8612	0.8665	0.8834
13	Huhehaote	0.8440	0.8093	0.8022	0.7931
14	Jinan	0.7322	1.0000	0.8009	1.0000
15	Kunming	1.0000	0.8223	0.8243	0.8605
16	Lanzhou	0.6286	0.6285	0.6348	0.6416
17	Lhasa	0.7042	0.7168	0.6770	0.6695
18	Nanchang	1.0000	0.9359	0.9413	1.0000
19	Nanjing	1.0000	0.9834	1.0000	0.9973
20	Nanning	1.0000	1.0000	1.0000	1.0000
21	Shanghai	1.0000	1.0000	1.0000	1.0000
22	Shenyang	0.8619	1.0000	0.7578	0.7071
23	Shijiazhuang	0.6198	0.6158	0.6266	0.6221
24	Taiyuan	0.6549	0.6498	0.6554	0.6629
25	Tianjin	1.0000	1.0000	0.9715	0.9698
26	Wuhan	0.8735	0.8238	0.8237	0.8281
27	Urumqi	0.7035	0.6951	0.6967	0.6891
28	Xian	1.0000	1.0000	0.8032	0.7667
29	Xining	0.6097	0.6128	0.6122	0.6194
30	Yinchuan	0.6740	0.6738	0.6735	0.6808
31	Zhengzhou	0.9516	1.0000	1.0000	1.0000

Changsha, Chengdu, Guiyang, Haikou, Hohhot, Wuhan, Xi’an, Kunming, and Hangzhou had falling GDP efficiencies, with Guiyang’s falling from 0.8144 in 2013 to 0.6585 in 2016. Xi’an attained 1 in both 2013 and 2014, but in the following two years, the GDP efficiency fell to 0.8032 and 0.7667.

Table 6 shows the CO_2_ emissions from 2013 to 2016, from which it can be seen that there were significant differences between the cities. Beijing, Chongqing, Guangzhou, Nanning and Shanghai had CO_2_ emissions efficiencies of 1 in all years, Chengdu, Haikou and Xi’an had CO_2_ emissions efficiencies of 1 in three years, and Changsha, Kunming, Lanzhou, Taiyuan, and Yinchuan had poor CO_2_ emissions efficiencies, with Taiyuan’s performance being the worst at only 0.3127.

**Table 6:** CO_2_ emissions efficiencies

No.	DMU	2013 CO_2_	2014 CO_2_	2015 CO_2_	2016 CO_2_
1	Beijing	1.0000	1.0000	1.0000	0.9999
2	Changchun	0.9501	0.9773	0.9631	0.9771
3	Changsha	0.5871	0.7055	0.7119	0.6986
4	Chengdu	1.0000	0.9587	1.0000	1.0000
5	Chongqing	1.0000	1.0000	1.0000	1.0000
6	Fuzhou	0.9019	1.0000	0.9783	0.9747
7	Guangzhou	1.0000	1.0000	1.0000	1.0000
8	Guiyang	0.5823	0.6059	1.0000	0.9156
9	Harbin	1.0000	1.0000	0.8694	0.8386
10	Haikou	0.9311	1.0000	1.0000	1.0000
11	Hangzhou	0.6579	0.7990	0.8613	0.8663
12	Hefei	0.7952	0.9847	0.9999	1.0000
13	Huhehaote	0.8094	0.4363	0.8417	0.8027
14	Jinan	0.6888	0.4811	0.5828	1.0000
15	Kunming	0.5492	0.6093	0.8413	0.8496
16	Lanzhou	0.5676	0.5757	0.4725	0.5080
17	Lhasa	0.9078	1.0000	0.9975	0.9999
18	Nanchang	0.9044	1.0000	0.9732	0.8400
19	Nanjing	0.5543	0.6039	0.8323	0.8223
20	Nanning	1.0000	1.0000	1.0000	1.0000
21	Shanghai	1.0000	1.0000	1.0000	1.0000
22	Shenyang	0.6833	0.5926	0.7766	0.9030
23	Shijiazhuang	0.7436	0.6944	1.0000	0.6940
24	Taiyuan	0.2691	0.2497	0.2607	0.3127
25	Tianjin	0.6306	0.6370	0.6483	0.6682
26	Wuhan	0.7127	0.8393	0.8543	0.8426
27	Wulumuqi	0.8574	0.8148	0.9301	0.7824
28	Xian	1.0000	0.9482	1.0000	0.9998
29	Xining	0.4210	0.4182	0.4427	0.9704
30	Yinchuan	0.4735	0.5294	0.3949	0.3796
31	Zhengzhou	1.0000	0.9102	0.9999	0.5149

Changsha, Fuzhou, Guiyang, Hangzhou, Hefei, Kunming, Lhasa, Tianjin and Xining Progress had rising CO_2_ emissions efficiencies with Hefei’s rising to 1 in 2015 and 2016, Kunming’s and Nanjing’s rising in 2015 and 2016, and Xining having a CO_2_ emissions efficiency of less than 0.5 in the first three years, but 0.9704 in 2016. However, the CO_2_ emissions efficiencies in Harbin, Urumqi and Zhengzhou fell; Zhengzhou’s efficiency was greater than 0.9 in the first three years but only 0.5149 in 2016.

The CO_2_ emissions efficiency improvements in most cities were significant, possibly because of the media reports and regulatory governance measures.

Table 7 shows the AQI efficiencies in the 31 Chinese cities from 2013 to 2016, from which it can be seen that the overall efficiencies in most cities were below 0.5.

**Table 7:** AQI efficiencies

No.	DMU	2013 AQI	2014 AQI	2015 AQI	2016 AQI
1	Beijing	1.0000	1.0000	1.0000	0.9545
2	Changchun	0.2447	0.3204	0.2647	0.3300
3	Changsha	0.2345	0.3802	0.3750	0.3910
4	Chengdu	1.0000	1.0000	0.5392	0.5384
5	Chongqing	1.0000	1.0000	1.0000	1.0000
6	Fuzhou	0.6379	0.6191	0.5834	0.5540
7	Guangzhou	1.0000	1.0000	1.0000	1.0000
8	Guiyang	0.1914	0.2634	0.3708	0.3168
9	Harbin	0.4610	0.5897	0.4827	0.5877
10	Haikou	0.2787	0.3596	0.3042	0.3070
11	Hangzhou	0.2570	0.5264	0.4464	0.4976
12	Hefei	0.1094	0.2396	0.2356	0.2441
13	Huhehaote	0.2182	0.1823	0.1603	0.1631
14	Jinan	0.3054	0.3339	0.4272	1.0000
15	Kunming	0.4146	0.4247	0.3997	0.3721
16	Lanzhou	0.2828	0.2132	0.1877	0.1546
17	Lhasa	0.0831	0.0732	0.0701	0.0595
18	Nanchang	0.1615	0.2230	0.2040	0.2392
19	Nanjing	0.2781	0.6482	0.3665	0.3919
20	Nanning	1.0000	1.0000	1.0000	1.0000
21	Shanghai	1.0000	1.0000	1.0000	1.0000
22	Shenyang	0.3846	0.4561	0.3357	0.3888
23	Shijiazhuang	0.3057	0.3464	0.4253	0.3270
24	Taiyuan	0.2875	0.2278	0.2110	0.1739
25	Tianjin	0.6446	0.5446	0.5353	0.5187
26	Wuhan	0.2862	0.4879	0.4537	0.4099
27	Urumqi	0.2149	0.1629	0.1496	0.2212
28	Xian	1.0000	0.7312	0.7551	0.4939
29	Xining	0.1838	0.1319	0.1277	0.1167
30	Yinchuan	0.1582	0.1375	0.1090	0.0963
31	Zhengzhou	0.3712	0.5058	0.3957	0.8236

The AQI efficiencies in Chongqing, Guangzhou, Nanning and Shanghai were 1 in all years and Beijing’s was 0.9545 in 2015, and 1 in all others years. However, few other cities had efficiencies greater than 0.8. Chengdu’s AQI efficiency was 1 in 2013 and 2014, but its 2015 and 2016 efficiencies were 0.5392 and 0.5384, and the AQI efficiencies in Changchun, Changsha, Guiyang, Haikou, Hefei, Hohhot, Lanzhou, Lhasa, Nanchang, Taiyuan, Urumqi, Xining and Yinchuan were all less than 0.4. Therefore, there was a significant need for AQI efficiency improvements in most cities.

Changchun, Changsha, Harbin, Hangzhou, Hefei, Jinan, Nanchang, Nanjing and Zhengzhou had rising AQI efficiencies, with Jinan’s rising from 0.3054 in 2013 to 1.0000 in 2016. However, Chengdu, Fuzhou, Hohhot, Lanzhou, Lhasa, Taiyuan, Tianjin, Xi’an, Xining, Yinchuan all had falling AQI efficiencies. Most cities were found to have poor AQI efficiencies, and there was insufficient evidence to determine the effects of the media reports.

### Media reports, CO_2_ and AQI analyses

Table 8 shows the media report, CO_2_ emissions, and AQI efficiencies in the 31 Chinese cities from 2013 to 2016. When the news media reports were considered as an input indicator, the AQI efficiencies were significantly lower than the CO_2_ emissions efficiencies. Shanghai, Nanning, Chongqing and Guangzhou had AQI efficiencies of 1, Chengdu had an AQI efficiency of 1 in 2013 and 2014, but suffered a significant decline in 2015 and 2016, Jinan’s AQI efficiency rose to 1 in 2016, and the other 24 cities had AQI efficiencies below 0.5.

**Table 8:** Media reports, CO_2_ and AQI efficiencies

No.	DMU	2013 Media	2014 Media	2015 Media	2016 Media	2013 CO_2_	2014 CO_2_	2015 CO_2_	2016CO_2_	2013AQI	2014AQI	2015AQI	2016AQI
1	Beijing	1	1	1	0.0888	1	1	1	0.9999	1	1	1	0.9545
2	Changchun	1	1	0.453	0.5095	0.9501	0.9773	0.9631	0.9771	0.2447	0.3204	0.2647	0.33
3	Changsha	0.7947	0.294	0.2613	0.3511	0.5871	0.7055	0.7119	0.6986	0.2345	0.3802	0.375	0.391
4	Chengdu	1	1	0.6895	0.7018	1	0.9587	1	1	1	1	0.5392	0.5384
5	Chongqing	1	1	1	1	1	1	1	1	1	1	1	1
6	Fuzhou	1	0.5194	0.2958	0.2476	0.9019	1	0.9783	0.9747	0.6379	0.6191	0.5834	0.554
7	Guangzhou	1	1	1	1	1	1	1	1	1	1	1	1
8	Guiyang	1	1	0.7661	0.6939	0.5823	0.6059	1	0.9156	0.1914	0.2634	0.3708	0.3168
9	Harbin	1	1	0.3266	0.3145	1	1	0.8694	0.8386	0.461	0.5897	0.4827	0.5877
10	Haikou	1	0.5106	0.3486	1	0.9311	1	1	1	0.2787	0.3596	0.3042	0.307
11	Hangzhou	0.7641	0.2972	0.1757	0.2636	0.6579	0.799	0.8613	0.8663	0.257	0.5264	0.4464	0.4976
12	Hefei	0.5486	0.2558	0.1693	0.161	0.7952	0.9847	0.9999	1	0.1094	0.2396	0.2356	0.2441
13	Huhehaote	1	0.6771	0.8171	0.6576	0.8094	0.4363	0.8417	0.8027	0.2182	0.1823	0.1603	0.1631
14	Jinan	0.123	0.2002	0.2545	1	0.6888	0.4811	0.5828	1	0.3054	0.3339	0.4272	1
15	Kunming	1	1	1	1	0.5492	0.6093	0.8413	0.8496	0.4146	0.4247	0.3997	0.3721
16	Lanzhou	0.3103	0.3244	0.2357	0.1733	0.5676	0.5757	0.4725	0.508	0.2828	0.2132	0.1877	0.1546
17	Lhasa	1	0.4733	0.3449	1	0.9078	1	0.9975	0.9999	0.0831	0.0732	0.0701	0.0595
18	Nanchang	0.4642	0.2935	0.16	0.3062	0.9044	1	0.9732	0.84	0.1615	0.223	0.204	0.2392
19	Nanjing	0.1958	0.2582	0.1836	0.167	0.5543	0.6039	0.8323	0.8223	0.2781	0.6482	0.3665	0.3919
20	Nanning	1	1	1	1	1	1	1	1	1	1	1	1
21	Shanghai	1	1	1	1	1	1	1	1	1	1	1	1
22	Shenyang	1	1	0.442	0.9326	0.6833	0.5926	0.7766	0.903	0.3846	0.4561	0.3357	0.3888
23	Shijiazhuang	0.3354	0.2719	0.2119	0.2302	0.7436	0.6944	1	0.694	0.3057	0.3464	0.4253	0.327
24	Taiyuan	0.6766	0.7599	0.663	0.4026	0.2691	0.2497	0.2607	0.3127	0.2875	0.2278	0.211	0.1739
25	Tianjin	1	0.7134	0.5127	0.5948	0.6306	0.637	0.6483	0.6682	0.6446	0.5446	0.5353	0.5187
26	Wuhan	1	0.506	0.3925	0.292	0.7127	0.8393	0.8543	0.8426	0.2862	0.4879	0.4537	0.4099
27	Urumqi	0.1846	0.2157	0.1367	0.3238	0.8574	0.8148	0.9301	0.7824	0.2149	0.1629	0.1496	0.2212
28	Xian	1	1	0.9544	1	1	0.9482	1	0.9998	1	0.7312	0.7551	0.4939
29	Xining	0.2904	0.367	0.2216	0.2259	0.421	0.4182	0.4427	0.9704	0.1838	0.1319	0.1277	0.1167
30	Yinchuan	0.3333	0.2819	0.2	0.1148	0.4735	0.5294	0.3949	0.3796	0.1582	0.1375	0.109	0.0963
31	Zhengzhou	0.4463	0.1225	0.0942	0.8728	1	0.9102	0.9999	0.5149	0.3712	0.5058	0.3957	0.8236

Therefore, the media news reports efficiencies in the cities with low AQI efficiencies required significant improvement. Hangzhou, Hefei, Nanchang, Nanjing, Shijiazhuang, Wuhan, Urumqi, Xining and Zhengzhou had CO_2_ emissions efficiencies higher than 0.8, but the CO_2_ emissions efficiencies in Changsha, Lanzhou, Taiyuan, Tianjin and Yinchuan were less than 0.8.

The media report efficiency fluctuations were found to be consistent with the AQI efficiency fluctuations, and the media reports in most cities had a more positive impact on CO_2_ emissions efficiencies than AQI efficiencies.

## Index efficiencies in each city and key areas for improvement

The input and output indicator efficiencies in each city from 2013 to 2016 were further examined to identify the needed improvements and are summarized in Table 9.

**Table 9:** Improvements in each city

No.	DMU	Improvement
1	Beijing	The 2016 media and AQI efficiencies need improvements
2	Changchun	Measures need to be taken for labor input efficiency, the news media efficiency also dropped significantly in the last two years, and there is a need to strengthen the AQI efficiency
3	Changsha	The media report, energy consumption, CO_2_ emissions and AQI efficiencies need significant improvements, with the most serious governance attention needed on AQI and then CO_2_ emissions
4	Chengdu	Both media report and AQI efficiencies need priority governance and the GDP efficiency also needs attention
5	Chongqing	There was no need for improvements as all input and output indicators were efficient
6	Fuzhou	Media efficiency dropped significantly, and there are also improvements needed in GDP efficiency. While the AQI efficiency had significant improvements, greater efforts are needed
7	Guangzhou	The improvement space for each indicator is 0
8	Guiyang	Media report efficiency fell, and energy efficiency rose. However, priority governance and focus are needed in GDP and AQI efficiencies, and CO_2_ emissions efficiency also needs attention
9	Harbin	Labor input efficiency is rising, but further governance is needed. The news media efficiency declined significantly in the last two years, even though the efficiency was fluctuating, significant AQI improvements are also needed,
10	Haikou	Media report efficiency had significant fluctuations, falling to a minimum in 2015, and then rising to 1, and while the GDP efficiency improved, more attention is needed
11	Hangzhou	As the media report efficiency dropped significantly, the room for improvement remained large. The AQI and CO_2_ emissions efficiencies need priority co-governance
12	Hefei	Media report efficiency declined, and labor input efficiency need improvements. Although the AQI efficiency slightly improved, the highest was only 0.24; therefore, there is still a great need for proper governance
13	Huhehaote	Priority needs to be given to vigorously managing AQI efficiency as the efficiency in 2016 was only 0.16. The media report efficiency fluctuated but still needs to be addressed and governed
14	Jinan	While the media report efficiency declined, and the AQI efficiency rose, both still need strong efficiency governance
15	Kunming	The media report efficiency continued to decline, and improvements should be prioritized, the energy consumption efficiency needs focused governance, the AQI efficiency score was slightly better than the media report efficiency and should also be given priority
16	Lanzhou	All indicators need improvements; priority governance should be given to the AQI and media report efficiencies, and there is also a need for energy consumption, CO_2_ emissions, AQI and GDP efficiency governance improvements
17	Lhasa	Media efficiency fluctuated and was the best in 2016; however, further improvements are needed. AQI and GDP efficiencies also need close governance attention
18	Nanchang	While there is less media report efficiency volatility, more improvements are needed. Further attention is needed to strengthen labor input efficiency governance, and while the AQI efficiency increased slightly, significant improvements are still needed
19	Nanjing	Media report efficiency declined significantly, and AQI efficiency fluctuated; therefore, governance in both areas needs to be strengthened
20	Nanning	There was no need for improvements as all input and output indicators were efficient
21	Shanghai	There was no need for improvements as all input and output indicators were efficient
22	Shenyang	The AQI efficiency is declining and priority governance is needed and while the CO_2_ emissions and energy consumption efficiencies continued to rise, effective governance needs to be maintained
23	Shijiazhuang	Media, energy consumption, and AQI efficiencies need continued governance
24	Taiyuan	GDP efficiency and especially energy consumption efficiency need priority attention, but some improvements are also needed in media reporting. Overall, the governance of AQI, and CO_2_ emissions efficiencies should be strengthened
25	Tianjin	Improvements are needed in the media, energy consumption, CO_2_ emissions, and AQI efficiencies. Therefore, better overall governance is needed
26	Wuhan	It is necessary to strengthen governance on the AQI and media report efficiencies
27	Urumqi	The media report and AQI efficiencies need priority governance, and the GDP and energy consumption efficiencies also need attention
28	Xian	The GDP efficiency declined in the last two years and the AQI efficiency also fell; therefore, governance needs to be strengthened in these two areas
29	Xining	AQI efficiency improvement needs priority governance, followed by GDP and media report efficiency, both of which declined. While energy consumption efficiency increased significantly in the last year, attention is still needed
30	Yinchuan	Priority management is needed for AQI efficiency, and media report efficiency also needs to be significantly improved. Further, as CO_2_ emissions and energy consumption efficiencies continued to decline, greater governance is needed
31	Zhengzhou	While media report efficiency rose significantly in 2016, continued attention is needed, and AQI efficiency also needs improvement

The differences in the regional urban characteristics and resource endowments have led to differences in industrial structures and production methods in each city, and the impact of media reports on CO_2_ emissions s and the AQI also varied widely, all of which meant that there were significant differences in the improvement needs. For example, in 2016, Beijing needed greater improvements in media reports and air quality efficiencies than in other years. Urumqi and Zhengzhou also need to place priority on improving media report and AQI efficiencies, Changsha and Taiyuan need AQI and CO_2_ emissions efficiency improvements, and Guiyang, Hohhot, Lanzhou, Lhasa, Shenyang, Xi’an and Xining all need to prioritize AQI and GDP efficiencies.

## Conclusions

This study used a modified undesirable dynamic DEA to study the energy consumption, environmental, and media report efficiencies in 31 Chinese cities, the conclusions from which were as follows.

First, Chongqing, Guangzhou, Nanjing, and Shanghai had overall efficiencies of 1, and the worst overall efficiencies were in Lanzhou, Shijiazhuang, Taiyuan, Xining and Yinchuan. The efficiencies in 12 cities declined, and the overall efficiencies in 5 cities fluctuated.

Second, Chongqing, Guangzhou, Kunming, Nanning and Shanghai had media report efficiencies of 1, Hefei, Nanchang, Nanjing, Urumqi, Xining, Yinchuan had the worst media report efficiencies, the efficiencies in Jinan and Urumqi increased, and the other 23 cities had reduced media report efficiencies.

Third, Beijing, Chongqing, Guangzhou, Nanning, and Shanghai had energy consumption efficiencies of 1, the energy consumption efficiencies in Taiyuan. Harbin, Lanzhou, Nanchang, Urumqi, Yunnan and Zhengzhou declined, and the energy consumption efficiencies in Changchun, Changsha, Fuzhou, Guiyang, Hangzhou, Hefei, Hohhot, Kunming, Nanjing, Shenyang, Taiyuan, Tianjin, Wuhan, and Xining increased.

Fourth, Beijing, Changchun, Guangzhou, Chongqing, Nanning, and Shanghai had GDP efficiencies of 1, Fuzhou, Shijiazhuang, Taiyuan, Urumqi, Xining, and Yinchuan’s had increasing GDP efficiencies, and Changsha, Chengdu, Guiyang, Haikou, Hohhot, Wuhan, Xi’an, Kunming, and Hangzhou had decreasing GDP efficiencies.

Fifth, Beijing, Chongqing, Guangzhou, Nanning and Shanghai had CO_2_ emissions efficiencies of 1, Changsha, Fuzhou, Guiyang, Hangzhou, Hefei, Kunming, Lhasa, Tianjin and Xining had increasing CO_2_ emissions efficiencies, Harbin, Urumqi and Zhengzhou’s had falling CO_2_ emissions efficiencies, and Taiyuan had the worst CO_2_ emissions efficiency.

Sixth, the overall AQI efficiencies in the 31 cities were low. While 13 cities had AQI efficiencies of 1, 10 cities had significant declines. No evidence was found to indicate that media reports had any impact on air pollution efficiency improvements.

## Policy implication

The governance focus, therefore, should be on improving the CO_2_ emissions and AQI efficiencies to prepare for the challenges of dealing with climate change and coordinating economic growth and environmental protection. Based on the above conclusions, the following management recommendations are given.

First, energy and industrial structural adjustments can improve the AQI efficiency. Of the 31 cities, at least 21 cities need AQI efficiency improvements, most of which have middle economic and social development levels, such as Changchun, Changsha, Fuzhou, Guiyang, Harbin, Hefei, Kunming, Lhasa, Nanchang, Nanjing, Shenyang, Shijiazhuang, Taiyuan, Wuhan, Urumqi, Xining, and Yinchuan. The economic growth and social development in these cities depend on traditional fossil fuel energy and traditional manufacturing and secondary industries that consume fossil energy. By adjusting their energy and industrial structures, the AQI and CO_2_ efficiencies could be improved.

Second, media report efficiency improvements need government support and encouragement. The media report content and effectiveness should be fully investigated and greater encouragement and support given to journalists and media outlets to encourage them to learn about environmental air protection and air pollution emissions and ensure media news report quality. At the same time, media workers need to keep their mission in mind and correctly guide the behavior of residents and businesses through active and accurate news reports. It is also necessary to actively play a supervisory role through media news reports to expose companies that violate regulations. As environmental awareness enhances the health of civilians and promotes economic growth, the news media needs to promote environmental protection and increase its environmental pollution coverage.

Third, news reports need to be timely when reporting on environmental issues, which also requires that government departments provide timely information to the public and the media. The news media could alert the public to environmental issues, which could assist in reducing the hazards or damage caused.

Fourth, cities needs to increase their green areas, forest coverage, and carbon sink capacities, and adjust their energy structures by developing new energy technologies to replace traditional fossil fuel based energy, all of which would improve CO_2_ emissions efficiency. Although the CO_2_ emissions efficiencies were found to be slightly better than the AQI index efficiencies, CO_2_ emissions needs better governance in Lanzhou, Shijiazhuang, Taiyuan and Yinchuan as the dependence in these cities on traditional fossil energy has affected their CO_2_ emissions efficiencies. Through measures such as greening and economic, industrial, and energy structural challenges, CO_2_ emissions can be effectively reduced.

Fifth, media reports can have positive impact on carbon-emissions-friendly lifestyles and consumption. Residents cannot understand the climate change challenges, air pollution hazards, or prevention and control technologies if they are given insufficient awareness of the environmental impact of their individual choices. Therefore, accurate media reports are needed to encourage positive environmentally friendly behavioral change and government needs to encourage the media to strengthen their attention on the impact of climate change to better guide behavioral and lifestyle changes.

Sixth, the government needs to adopt environmentally focused market and fiscal policies, such as a carbon trading market, to encourage companies to choose environmentally friendly behaviors, implement positive behaviors, and fulfill their environmental and social responsibilities. Even though these cities have different industrial and energy structures, they need to develop and utilize new energy in accordance with local conditions to replace traditional fossil fuels. A carbon trading market could therefore assist in balancing economic development and environmental protection. Finally, the government needs to coordinate CO_2_ and other air pollutants with all stakeholders such as news media, residents, and enterprises to integrate resources and reduce costs. The media, residents, enterprises, scientific research institutions, schools, and non-government organizations need to work in harmony to reduce CO_2_ and other air pollutant emissions.

## Limitation and further research directions

This research was focused on achieving atmospheric environmental efficiency in 31 Chinese cities to achieve effective CO_2_ and air pollutant emissions reductions, and therefore has strong practical significance and value. Due to time constraints and insufficient access to information, this study only used data from 2013 to 2016; therefore, the data needs to be updated in future research. In addition to time series data, new research could also divide the news media data to more fully examine the changes over time.

## Data Availability

Not applicable.
